# Prevalence and correlates of non-disclosure of maternal HIV status to male partners: a national survey in Kenya

**DOI:** 10.1186/s12889-018-5567-6

**Published:** 2018-05-30

**Authors:** John Kinuthia, Benson Singa, Christine J. McGrath, Beryne Odeny, Agnes Langat, Abraham Katana, Lucy Ng’ang’a, Jillian Pintye, Grace John-Stewart

**Affiliations:** 1 Kenyatta National Hospital, P.O. Box 2590-00202, Nairobi, Kenya; 2 Kenya Medical Research Institute, Nairobi, Kenya; 3 University of Washington, Seattle, WA USA; 4 Division of Global HIV & TB, US Centers for Disease Control and Prevention (CDC), Nairobi, Kenya

**Keywords:** HIV, Male partner, Non-disclosure, Antiretrovirals, PMTCT

## Abstract

**Background:**

Prevention of mother-to-child HIV transmission (PMTCT) programs usually test pregnant women for HIV without involving their partners. Non-disclosure of maternal HIV status to male partners may deter utilization of PMTCT interventions since partners play a pivotal role in decision-making within the home including access to and utilization of health services.

**Methods:**

Mothers attending routine 6-week and 9-month infant immunizations were enrolled at 141 maternal and child health (MCH) clinics across Kenya from June–December 2013. The current analysis was restricted to mothers with known HIV status who had a current partner. Multivariate logistic regression models adjusted for marital status, relationship length and partner attendance at antenatal care (ANC) were used to determine correlates of HIV non-disclosure among HIV-uninfected and HIV-infected mothers, separately, and to evaluate the relationship of non-disclosure with uptake of PMTCT interventions. All analyses accounted for facility-level clustering,

**Results:**

Overall, 2522 mothers (86% of total study population) met inclusion criteria, 420 (17%) were HIV-infected. Non-disclosure of HIV results to partners was higher among HIV-infected than HIV-uninfected women (13% versus 3% respectively, *p* < 0.001). HIV-uninfected mothers were more likely to not disclose their HIV status to male partners if they were unmarried (adjusted odds ratio [aOR] = 3.79, 95% CI: 1.56–9.19, *p* = 0.004), had low (≤KSH 5000) income (aOR = 1.85, 95% CI: 1.00–3.14, *p* = 0.050), experienced intimate partner violence (aOR = 3.65, 95% CI: 1.84–7.21, p < 0.001) and if their partner did not attend ANC (aOR = 4.12, 95% CI: 1.89–8.95, *p* < 0.001). Among HIV-infected women, non-disclosure to male partners was less likely if women had salaried employment (aOR = 0.42, 95%CI: 0.18–0.96, *p* = 0.039) and each increasing year of relationship length was associated with decreased likelihood of non-disclosure (aOR = 0.90, 95% CI: 0.82–0.98, *p* = 0.015 for each year increase). HIV-infected women who did not disclose their HIV status to partners were less likely to uptake CD4 testing (aOR = 0.32, 95% CI: 0.15–0.69, *p* = 0.004), to use antiretrovirals (ARVs) during labor (OR = 0.38, 95% CI 0.15–0.97, *p* = 0.042), or give their infants ARVs (OR = 0.08, 95% CI 0.02–0.31, *p* < 0.001).

**Conclusion:**

HIV-infected women were less likely to disclose their status to partners than HIV-uninfected women. Non-disclosure was associated with lower use of PMTCT services. Facilitating maternal disclosure to male partners may enhance PMTCT uptake.

## Background

Worldwide, HIV testing among pregnant women has increased substantially in the last decade [1]. Pregnant women usually receive HIV testing during routine antenatal care, which is typically attended without male partners. Following testing, women are expected to disclose their HIV status to their male partners and encourage them to seek HIV testing [2]. World Health Organization (WHO) guidelines recommend couple HIV counseling and testing which provides an opportunity to test the woman together with her partner and enables provider-facilitated disclosure, counseling on safer sex practices and linkage to care if either partner is HIV-infected [3, 4]. However, low male partner antenatal clinic (ANC) attendance in sub-Saharan Africa limits the utility of clinic-based couple testing approaches as a strategy to facilitate disclosure [5].

Approaches to promote male partner involvement such as reorganizing ANC clinic operations to offer male testing during evenings or weekends, fast tracking pregnant women who are accompanied by their male partners and written invitations to male partners to attend ANC have not consistently resulted in marked increase in number of men attending ANC clinics [6–8]. Alternative strategies include home-based testing which have been shown to reach more male partners and results in increased couple HIV testing and facilitated mutual disclosure [9, 10].

A systematic review in 2014 reported rates of disclosure of HIV status among pregnant and postpartum women in sub-Saharan African vary widely, ranging from 30.2 to 93.3% [11]. HIV-uninfected women have few or no concerns about disclosing their HIV status to their partners [12]. In contrast, HIV-infected women may delay disclosure or choose not to disclose due to fears of accusations of infidelity, abandonment, discrimination, and violence [12, 13]. Previous studies from Kenya found maternal disclosure of HIV status to male partners was associated with use of antiretroviral drugs for PMTCT [14], higher rates of facility delivery [14], and adherence to infant feeding guidance [15] thereby reducing risk of vertical HIV infection and increasing infant HIV free survival [16]. However, correlates of non-disclosure are not well defined. As PMTCT Option B+ scales up with regimens that require life-long antiretroviral treatment (ART) adherence, the need for HIV-infected pregnant and breastfeeding women to disclose their status to their male partners becomes more critical in order to maintain adherence to ART. In a national survey evaluating effectiveness of PMTCT programs in Kenya, we assessed prevalence and correlates of maternal non-disclosure of HIV status to male partners and impact on utilization of PMTCT services.

## Methods

### Study design

The methodology of the parent study has been described previously [17]. Briefly, we conducted two facility-based cross-sectional surveys of PMTCT effectiveness from June to December 2013. The first, PMTCT-MCH survey evaluated the population-level effectiveness of the national PMTCT program among all women attending randomly selected facilities in seven of eight provinces in Kenya. The second Nyanza oversample survey purposively sampled HIV-infected women attending facilities in Nyanza, a former province with the highest HIV prevalence in Kenya [18].

### Facility selection

The PMTCT-MCH survey used probability proportionate to size sampling to randomly sample 120 facilities from among the 540 medium and large facilities across Kenya. The Nyanza Oversample survey included all large facilities in the former Nyanza province (*n* = 30). Nine facilities in the former Nyanza were included in both surveys, thus a total of 141 facilities were sampled between both surveys. Facilities located in the North Eastern province were excluded due to security concerns and logistic feasibility.

### Study population

All mothers bringing their infants for 6-week or 9-month infant immunizations were eligible to participate. The National PMTCT-MCH survey recruited all eligible mother-infant pairs attending selected facilities during a fixed 5-day recruitment period, regardless of maternal HIV status. The Nyanza Oversample survey recruited all eligible HIV-positive mothers and their infants attending selected facilities in Nyanza during a fixed 10-day recruitment period. Mothers were included in the current analysis if they had data available on HIV status and reported a current male partner.

### Data collection

Study staff administered the survey using Open Data Kit on tablet computers. The survey instrument was adapted from previous surveys designed to measure PMTCT effectiveness [19–21], and field tested prior to implementation. The questionnaire included uptake of ANC, maternal HIV testing, non-disclosure of status, partner HIV status, intimate partner violence (IPV), and use of ARVs among HIV-infected women as well as maternal and paternal demographics and reproductive and family planning history. Among HIV exposed infants, ARVs and HIV testing were assessed. IPV was defined by a score ≥ 10.5 on the Hurt Insult Threaten Scream (HITS) scale [22].

### Statistical analyses

Statistical models were analyzed separately for HIV-infected and uninfected women to describe the study population and examine the correlates of non-disclosure in these two unique groups. All analyses accounted for facility-level clustering. We determined correlates of non-disclosure of HIV status and impact of non-disclosure on utilization of PMTCT services using logistic regression models. Multivariate logistic regression was conducted for covariates statistically associated (*p* < 0.05) with non-disclosure in univariate analysis. We decided a priori to adjust all multivariate models for marital status, relationship length and male partner attendance at ANC based on previous literature which identified relationship stability and partner engagement in care as predictors of disclosure [23]. STATA version 11 (STATA Corp, College Station, Texas, USA) was used to analyze data.

## Results

Overall, 2522 mothers (86% of total study population) had known HIV status, reported having a current partner and were included in the final analysis. Of these mothers, 420 (17%) were HIV-infected and 2102 (83%) were HIV-uninfected. The mean age was 28.4 years (standard deviation [SD] 5.5) for HIV-infected mothers and 25.8 years (SD 5.5) for HIV uninfected mothers. Ninety-seven percent of both HIV-infected and HIV-uninfected mothers were married or cohabiting with their current parent; the mean relationship duration was 6.8 years (SD 5.2) and 5.3 years (SD 4.7) for HIV-infected and HIV-uninfected mothers, respectively. Among HIV-infected women, frequency of having no formal education was 8 and 9% had salaried employment; 7% of HIV-uninfected women had no formal education and 11% had salaried employment (Table 1).

**Table 1:** Characteristics of mothers with current male partners and known HIV status (*n* = 2522)^a^

Characteristic		*N* (%), Mean (SD)	
		HIV-infected (*n* = 420)	HIV-uninfected (*n* = 2102)
*Maternal characteristics*			
Age (years)		28.4 (5.5)	25.8 (5.5)
Unmarried/not cohabiting		11 (3%)	64 (3%)
Duration of relationship (years)		6.8 (5.2)	5.3 (4.7)
Education	None	32 (7.6%)	141 (6.7%)
	Primary	243 (58%)	988 (47%)
	Secondary	111 (26%)	669 (32%)
	> Secondary	34 (8%)	304 (14%)
Employment	Salaried	38 (9%)	221 (11%)
	Self employed	138 (33%)	564 (27%)
	Housewife	173 (41%)	956 (46%)
	Unemployed	68 (16%)	335 (16%)
Income/month	KSH ≤ 5000	118 (48%)	837 (62%)
	KSH > 5000	128 (52%)	507 (38%)
IPV (from current male partner)^b^		60 (14%)	111 (5%)
Current male partner characteristics			
Partner age		35.0 (7.7)	31.2 (6.8)
Partner education	None	19 (5%)	63 (3%)
	Primary	150 (39%)	697 (34%)
	Secondary	175 (45%)	859 (42%)
	> Secondary	44 (11%)	405 (20%)
Partner employment	Unemployed	54 (13%)	240 (12%)
	Self employed	199 (48%)	943 (45%)
	Salaried	162 (39%)	900 (43%)
Partner tested for HIV		322 (77%)	1471 (70%)
Partner HIV status^c^	HIV infected	228 (71%)	7 (1%)
	HIV uninfected	88 (27%)	1433 (97%)
	Unknown	6 (2%)	31 (2%)
Obstetric history			
Number of living children		2.9 (1.4)	2.3 (1.5)
Attended any ANC^d^		414 (99%)	2080 (99%)
≥4 ANC visits^d^		240 (59%)	988 (49%)
Partner did not attend ANC^d^		270 (66%)	1288 (62%)
Health facility delivery^d^		352 (84%)	1640 (78%)
HIV status disclosure			
Did not disclose HIV status to current male partner		53 (13%)	72 (3%)

*SD* standard deviation, *IPV* intimate partner violence, *ANC* antenatal care

^a^Missing data not shown, all models adjusted for clinic-level clustering

^b^IPV defined as having a score ≥ 10.5 on HITS scale

^c^Among male partners who were tested for HIV

^d^During the most recent pregnancy

Male partners of HIV-infected women had a mean age of 35.0 years (SD 7.7) and male partners of HIV-uninfected women had a mean age 31.2 years (SD 6.8). Few male partners of HIV-infected women (5%) and HIV-uninfected women (3%) had no education and frequency of unemployment among male partners was 13 and 12% for HIV-infected and HIV-uninfected mothers, respectively. Among mothers with male partners who had been tested for HIV, 71% of HIV-infected mothers and 1% of HIV-uninfected mothers had HIV-infected partners.

Overall, 125 of the 2522 (5%) mothers included in the analysis reported non-disclosure of HIV status to their partners. The proportion of HIV-infected women who did not disclose their status was significantly higher 13% (53/420) compared to 3% (72/2102) among HIV-uninfected women (*p* < 0.001).

### Utilization of maternal health services

Frequency of having the recommended ≥4 ANC was 59% among HIV-infected mothers and 49% among HIV-uninfected mothers. Approximately 1 in 3 male partners were reported to have attended antenatal care among both HIV-infected and uninfected mothers (Table 1). Delivering at health facility for their most recent birth was common among HIV-infected mothers (84%) and HIV-uninfected mothers (78%).

### Correlates of HIV non-disclosure among HIV-uninfected mothers

In univariate analyses, HIV-uninfected mothers were more likely not to disclose if they were unmarried, had an income ≤KSH 5000/month, and experienced IPV from their male partner or if their male partner did not attend ANC; delivery within a health facility was associated with decreased likelihood of non-disclosure (Table 2). All potential predictors significantly associated with non-disclosure among HIV-uninfected mothers in univariate models remained significant in multivariate models after adjustment for marital status, relationship length and male partner ANC attendance.

**Table 2:** Correlates of HIV status non-disclosure among HIV-uninfected mothers with current male partners (*n* = 2102)^a^

		Univariate		Multivariate^b^	
Characteristic		OR (95% CI)	*p*-value	aOR (95% CI)^2^	*p*-value
Maternal characteristics					
Age (years)		0.98 (0.93–1.03)	0.502		
Unmarried/not cohabiting		5.90 (2.69–12.94)	< 0.001*	3.79 (1.56–9.19)	0.004
Duration of relationship (years)		0.99 (0.94–1.04)	0.613		
Education	None	ref			
	Primary	1.67 (0.56–4.95)	0.350		
	Secondary	0.79 (0.25–2.46)	0.677		
	> Secondary	0.81 (0.23–2.86)	0.738		
Employment	Salaried	ref			
	Self employed	3.09 (0.93–10.31)	0.066		
	Housewife	2.35 (0.74–7.50)	0.146		
	Unemployed	3.64 (0.96–13.81)	0.057		
KSH ≤ 5000 income/month		2.25 (1.23–4.12)	0.009*	1.85 (1.00–3.41)	0.050
IPV (from current male partner)^c^		3.48 (1.83–6.62)	< 0.001*	3.65 (1.84–7.21)	< 0.001
Current male partner characteristics					
Partner age (years)		0.99 (0.94–1.04)	0.634		
Partner education	None	Ref			
	Primary	2.89 (0.38–21.99)	0.304		
	Secondary	1.71 (0.21–13.72)	0.613		
	> Secondary	1.25 (0.14–10.77)	0.838		
Partner employment	Unemployed	Ref			
	Self employed	1.55 (0.65–3.71)	0.321		
	Salaried	0.80 (0.30–2.10)	0.641		
Partner tested for HIV		1.25 (0.67–2.31)	0.476		
Partner HIV status^d^	HIV-uninfected	Ref			
	HIV-infected	–			
	Unknown	4.23 (0.92–19.50)	0.064		
Obstetric history					
Number of living children		1.08 (0.94–1.23)	0.266		
≥4 ANC visits ^e^		0.63 (0.40–0.99)	0.047		
Partner did not attend ANC^e^		4.17 (1.92–9.05)	< 0.001	4.12 (1.89–8.95)	< 0.001
Health facility delivery^e^		0.43 (0.26–0.71)	0.001*	0.47 (0.26–0.82)	0.009

*SD* standard deviation, *IPV* intimate partner violence, *ANC* antenatal care, *OR* odds ratio, *aOR* adjusted odds ratio

^a^Missing data not shown; all models adjusted for clinic-level clustering

^b^Multivariate models adjusted for marital status, duration of relationship and partner ANC attendance

^c^IPV defined as having a score ≥ 10.5 on HITS scale

^d^Among male partners who were tested for HIV

^e^During the most recent pregnancy

In multivariate analyses, the likelihood of non-disclosure was 4-fold higher among unmarried mothers compared to HIV-uninfected mothers who were married or cohabiting (adjusted odds ratio (aOR = 3.79, 95% CI: 1.56–9.19, *p* = 0.004). HIV-uninfected mothers who experienced IPV were nearly 4 times as likely to not disclose as mothers who did not experience IPV (aOR = 3.65, 95% CI: 1.84–7.21, *p* < 0.001). Having a partner who did not attend ANC was associated with a 4-fold higher likelihood of non-disclosure compared to having a partner who attended ANC among HIV-uninfected mothers (aOR = 4.12, 95% CI: 1.89–8.95, *p* < 0.001). HIV-uninfected mothers who delivered in healthcare facilities were less likely to not disclose their HIV status (aOR = 0.47, 95% CI: 0.26–0.82, *p* = 0.009).

### Correlates of HIV non-disclosure among HIV-infected mothers

In univariate analyses, HIV-infected mothers were more likely not to disclose their HIV status to their partners if they were unemployed or if their male partner did not attend ANC (Table 3). HIV-infected mothers were less likely to not disclose if they were in longer relationships and had employed partners in univariate analyses (Table 3). After adjustment for marital status, relationship length and male partner ANC attendance, all potential predictors significantly associated with non-disclosure among HIV-infected mothers in univariate models remained significant in multivariate models. No association was detected between non-disclosure and IPV, income or delivering in a health facility among HIV-infected mothers.

**Table 3:** Correlates of HIV status non-disclosure among HIV-infected mothers with current male partners (*n* = 420)^a^

		Univariate		Multivariate^b^	
Characteristic		OR (95% CI)	*p*-value	aOR (95% CI)^b^	*p*-value
Maternal characteristics					
Age (years)		0.95 (0.89–1.02)	0.137		
Unmarried/not cohabiting		1.56 (0.33–7.41)	0.572		
Duration of relationship (years)		0.91 (0.84–0.98)	0.017*	0.90 (0.82–0.98)	0.015*
Education	None	ref			
	Primary	1.31 (0.40–4.30)	0.653		
	Secondary	1.63 (0.46–5.78)	0.447		
	> Secondary	1.67 (1.41–6.74)	0.470		
Employment	Salaried	ref		ref	
	Self employed	4.18 (0.49–35.51)	0.188	4.11 (0.47–35.77)	0.198
	Housewife	5.67 (0.71–45.23)	0.100	5.07 (0.65–39.52)	0.120
	Unemployed	10.47 (1.20–91.31)	0.034*	9.19 (1.06–79.41)	0.044
KSH ≤ 5000 income/month		0.61 (0.26–1.47)	0.269		
IPV (from current male partner)^c^		1.08 (0.49–2.38)	0.853		
Current male partner characteristics					
Partner age (years)		0.97 (0.92–1.02)	0.209		
Partner education	None	ref			
	Primary	0.29 (0.08–1.03)	0.056		
	Secondary	0.40 (0.11–1.45)	0.163		
	> Secondary	0.44 (0.10–2.03)	0.290		
Partner employment	Unemployed	ref		ref	
	Self employed	0.59 (0.28–1.25)	0.165	0.61 (0.30–1.24)	0.169
	Salaried	0.43 (0.20–0.93)	0.033*	0.42 (0.18–0.96)	0.039
Partner tested for HIV		0.69 (0.26–1.81)	0.445		
Partner HIV status^d^	HIV-uninfected	ref			
	HIV-infected	0.46 (0.17–1.24)	0.124		
	Unknown	–			
Obstetric history					
Number of living children		0.89 (0.69–1.16)	0.405		
≥4 ANC visits^e^		0.65 (0.37–1.15)	0.139		
Partner did not attend ANC^e^		2.65 (1.29–5.43)	0.009*	2.62 (1.28–5.35)	0.009*
Health facility delivery^e^		1.10 (0.48–2.54)	0.823		
HIV-related characteristics					
CD4 testing uptake		0.29 (0.14–0.59)	0.001*	0.32 (0.15–0.69)	0.004
CD4 results received		0.49 (0.22–1.10)	0.084		
Currently on HAART for own health		0.39 (0.21–0.74)	0.005*	0.51 (0.25–1.04)	0.063
Maternal ARV use					
During pregnancy		0.42 (0.16–1.10)	0.078		
During labour		0.25 (0.11–0.53)	< 0.001*	0.38 (0.15–0.97)	0.042
During breastfeeding		0.30 (0.15–0.62)	< 0.001*	0.50 (0.22–1.14)	0.096
Infant received PCR testing		0.56 (0.31–1.01)	0.053*	0.60 (0.32–1.09)	0.093
Infant ARV use		0.08 (0.03–0.22)	< 0.001*	0.08 (0.02–0.31)	< 0.001

*SD* standard deviation, *IPV* intimate partner violence, *ANC* antenatal care, *OR* odds ratio, *aOR* adjusted odds ratio

^a^Missing data not shown; all models adjusted for clinic-level clustering

^b^Multivariate models adjusted for marital status, relationship length, and partner ANC attendance

^c^IPV defined as having a score ≥ 10.5 on HITS scale

^d^Among male partners who were tested for HIV

^e^During the most recent pregnancy

*highlights factors were significantly different in univariate analysis

In multivariate models among HIV-infected women, each increasing year of relationship duration was associated with a decreased likelihood of non-disclosure of HIV status to male partners (aOR = 0.90 per increase in year, 95%CI: 0.82–0.98, *p* = 0.015). Having salaried employed was associated with increased likelihood of non-disclosure compared to being unemployed among HIV-infected mothers (aOR = 9.19, 95% CI: 1.06–79.41, *p* = 0.044). Compared to HIV-infected mothers whose partners attended ANC, those with partners who did not attend were nearly 3 times more likely to not disclose (aOR = 2.62, 95% CI 1.28–5.35, *p* = 0.009). HIV-infected mother whose partners had salaried employment were less likely to not disclose than women whose partners were unemployed (aOR = 0.42, 95% CI 0.18–0.96, *p* = 0.039).

### Non-disclosure of HIV status and utilization of PMTCT services among HIV-infected mothers

Among HIV-infected mothers, those who did not disclose their HIV status to male partners were less likely to not uptake CD4 testing (aOR = 0.32, 95% CI: 0.15–0.69, *p* = 0.004), less likely to use ARVs during birth (aOR 0.38, 95% CI 0.15–0.97, *p* = 0.042) and less likely to give their infants ARVs (aOR = 0.08, 95% CI: 0.02–0.31, *p* < 0.001) compared to mothers who disclosed. There were trends towards an association between non-disclosure and decreased likelihood of using HAART (aOR = 0.51, 95% CI 0.25–1.04, *p* = 0.063), using ARVs during breastfeeding (aOR = 0.50, 95% CI: 0.22–1.14, *p* = 0.096) and receipt of infant PCR testing (aOR = 0.60, 95% CI: 0.32–1.09, *p* = 0.093), though our statistically power was limited to detect associations.

## Discussion

Even though most mothers (approximately 95%) reported disclosing their HIV status to their male partners in this survey of 141 MCH clinics in Kenya, HIV-infected mothers less frequently reported disclosure than HIV-uninfected mothers. The non-disclosure rate of 13% among HIV-infected mothers that we observed, although markedly higher than among HIV-uninfected mothers in the same clinics, is lower than in previous studies in Kenya and sub-Saharan Africa [13, 15, 24]. Two recent studies in Kenya from 2013 and 2014 reported that 51 and 69%, respectively, of pregnant HIV-infected mothers did not disclose their status [15, 24]. The lower levels of non-disclosure noted in our study may be due to sustained campaigns promoting HIV testing following launch of the elimination of mother-to-child transmission of HIV (EMTCT) and Keeping Mothers Alive Campaign in November 2012, wider knowledge about routinized HIV testing in pregnancy and increased availability and use of ART in recent years [25–27]. It is possible that there has been some decrease in stigma regarding HIV because of these developments that have encouraged pregnant HIV-infected women to disclose their status. However, despite these efforts, 1 in 8 HIV-infected mothers did not disclose their status and non-disclosure was associated with decreased uptake of PMTCT services. In our study, we found that non-disclosure of HIV status to male partners persists as a gap to maximizing improved health outcomes for mothers, their male partners and infants.

In sub-Saharan Africa, most HIV infections are acquired through heterosexual partnerships [28]. Women who test HIV-positive may therefore experience anxiety when considering disclosure due to fears of accusations of bringing HIV infection into the family through extramarital partnerships or promiscuity [12]. While it may be acceptable for a man to have more than one partner in some African communities, it is a taboo for women to do so and likely to provoke negative consequences [29–31]. Women who test HIV-positive fear that disclosure could result in abandonment, loss of economic security, discrimination and violence [13, 32–34]. Some HIV-infected women report encouragement or more kindness from their male partners following disclosure of status, but this is uncommonly reported [12]. Several studies have noted that disclosure remains extremely difficult for HIV-infected women and highlights the need to strengthen support services for these women in order to maximize opportunities for HIV prevention [12, 35].

Consistent with previous studies, we found that shorter duration of relationship and employment status were associated with non-disclosure of HIV-positive status, though our estimates for the association between employment and non-disclosure should be interpreted with caution due to imprecise estimations [34, 36]. Concerns about abandonment or breakdown of relationship have been cited as barriers to disclosure [13]. HIV-infected women in a relationship for a short duration may perceive that the partnership as weak and possibly not able to withstand the strain that would result from HIV disclosure. Women who are unemployed and those dependent on male partners for financial support may potentially opt not to disclose for fear that partners may discontinue support, particularly during pregnancy when it is more difficult for the women to find employment to support themselves. Strategies to empower these women are therefore an important complement in HIV prevention efforts.

Consistent with other studies we found that women who were unmarried were more likely not to disclose [34, 36, 37]. This association was seen in HIV-uninfected but not HIV-infected women. Unmarried women may have felt reassured by their HIV negative status and did not feel need to disclose status. However, in view of the high HIV discordance rates in Kenya, non-disclosure results in missed opportunity to encourage male partner testing as a strategy to identify couples in HIV discordant relationship and promote adoption of primary HIV prevention interventions among HIV-uninfected women [18].

We found that partner ANC non-attendance was associated with non-disclosure among both HIV-infected and HIV-uninfected mothers. Partner ANC attendance provides an opportunity to counsel and educate men about HIV and PMTCT, offer couple HIV testing and facilitate disclosure of HIV test results. However, male ANC attendance remains low in sub-Saharan African [5]. Low ANC attendance could possibly be due to historical view of reproductive health as analogous to women’s health and therefore almost exclusively the responsibility of the women [35]. Increasing male participation requires re-organization of services to be more welcoming for men and education campaigns to change beliefs and attitudes of men as well as addressing institutional barriers such as long wait times at the clinics [38, 39].

HIV-uninfected mothers who reported frequent IPV were more likely not to disclose. Surprisingly, this association was not seen among HIV- infected women. A recent meta-analysis evaluating risk factors of IPV among pregnant women in Africa reported a positive association between HIV infection and IPV [40]. A study in Kenya reported that after disclosure of HIV test results, the odds of HIV-positive pregnant women reporting domestic violence were 4.8 times those of HIV-negative women [41]. Additionally, a strong relationship has been reported between history of violence and current violence in pregnancy highlighting the need for routine screening for IPV during antenatal care. Similar to other studies, we did not find association between education level and disclosure [34, 37, 42]. Also, we did not find association between non-disclosure and partner education level, in contrast to a South African study which found that women with partners who had tertiary education were more likely to disclose [42].

Consistent with previous studies, we found that HIV-infected women who had not disclosed their results were less likely to use PMTCT services [43]. These findings explain our previous finding of higher rates of MTCT among women who have not disclosed status to their male partners [44]. Optimal utilization of PMTCT interventions can reduce the risk of vertical HIV transmission to below 5%, the target for EMTCT [45]. However, women who have not disclosed their status may have serious challenges with both uptake and adherence to maternal and or infant ARVs or exclusive breastfeeding, compromising efforts towards EMTCT [15, 46–48]. Our findings highlight the need for innovative strategies to facilitate utilization of available PMTCT interventions by women hesitant to disclose their status.

We found that of the HIV-infected women who had not disclosed their status, approximately 20% reported HIV-uninfected partners who were at risk of HIV acquisition. A modeling study among adults in Zambia and Rwanda estimated that between 55·1% and 92·7% of new heterosexually acquired HIV infections occurred within serodiscordant marital or cohabiting relationships [28]. Non-disclosure of status limits utilization of HIV preventive interventions such as condoms and pre-exposure prophylaxis [49, 50].

The study had several strengths and limitations. The study enrolled women from across Kenya, except for the former North Eastern Province, where access was limited due to security concerns. However, the region is sparsely populated limiting the impact of excluding this region. We enrolled both HIV-infected and uninfected women allowing for assessment of association of HIV status with non-disclosure. The relatively large sample size ensured that we had adequate power to assess correlates of non-disclosure. Small clinics were excluded and thus our estimates are not representative of small facilities. Oversampling women in Nyanza province allowed for an adequate number of HIV-infected women to assess correlates of non-disclosure among HIV-infected women. Limitations of the study included, recall bias in ascertainment of non-disclosure status, and reliance on self-report for HIV disclosure status, introducing the risk of social desirability bias. The study was not originally designed to primarily assess non-disclosure limiting the depth of our exploration of correlates. Finally, women were selected based on attendance at infant immunizations at participating health facilities, and thus this sample does not include the subset of potentially most at-risk women who do not attend MCH clinic.

## Conclusion

In conclusion, we found low rates of non-disclosure of maternal HIV status among all mothers, but higher among those who were HIV-infected. Non-disclosure among HIV-infected women was associated with reduced use PMTCT services. Promoting male partner antenatal clinic attendance may be useful to enhance disclosure and optimize PMTCT intervention adherence. There is need for novel strategies to facilitate use of PMTCT intervention by women reluctant to disclose their status.

## Abbreviations

ANC: Antenatal care
ART: Antiretroviral treatment
EMTCT: Elimination of mother-to-child transmission of HIV
IPV: Intimate partner violence
MCH: Maternal and child health
PMTCT: Prevention of mother-to-child HIV transmission
WHO: World Health Organization

## References

[1] UNAIDS. Global report: UNAIDS report on the global AIDS epidemic 2013*.* 2013; Available from: http://www.unaids.org/en/media/unaids/contentassets/documents/epidemiology/2013/gr2013/UNAIDS_Global_Report_2013_en.pdf.

[2] National AIDS and STI Control Programme, M.o.P.H.a.S., Kenya, *Guidelines for HIV Testing and Counselling in Kenya NASCOP;* 2008*.* 2008.

[3] Painter TM (2001). Voluntary counseling and testing for couples: a high-leverage intervention for HIV/AIDS prevention in sub-Saharan Africa. Soc Sci Med.

[4] World Health Organisation. *Guidance on couples HIV testing and counselling including antiretroviral therapy for treatment and prevention in serodiscordant couples: recommendations for a public health*. 2012; Available from: http://www.who.int/hiv/pub/guidelines/9789241501972/en/. 23700649

[5] Msuya SE (2008). Low male partner participation in antenatal HIV counselling and testing in northern Tanzania: implications for preventive programs. AIDS Care.

[6] Krakowiak D (2016). Home-based HIV testing among pregnant couples increases partner testing and identification of Serodiscordant partnerships. JAIDS J Acquir Immune Defic Syndr.

[7] Masters SH (2016). Promoting partner testing and couples testing through secondary distribution of HIV self-tests: a randomized clinical trial. PLoS Med.

[8] Onyango OA (2014). Home visits during pregnancy enhance male partner HIV counselling and testing in Kenya: a randomized clinical trial. AIDS.

[9] Osoti AO (2014). Home visits during pregnancy enhance male partner HIV counselling and testing in Kenya: a randomized clinical trial. AIDS.

[10] Doherty T, et al. Effect of home based HIV counselling and testing intervention in rural South Africa: cluster randomised trial. BMJ. 2013;346 10.1136/bmj.f3481. 10.1136/bmj.f3481 PMC3685510 23766483

[11] Tam M, Amzel A, Phelps BR (2015). Disclosure of HIV serostatus among pregnant and postpartum women in sub-Saharan Africa: a systematic review. AIDS Care.

[12] Rujumba J (2012). "Telling my husband I have HIV is too heavy to come out of my mouth": pregnant women's disclosure experiences and support needs following antenatal HIV testing in eastern Uganda. J Int AIDS Soc.

[13] Medley A (2004). Rates, barriers and outcomes of HIV serostatus disclosure among women in developing countries: implications for prevention of mother-to-child transmission programmes. Bull World Health Organ.

[14] Spangler SA (2014). HIV-positive status disclosure and use of essential PMTCT and maternal health Services in Rural Kenya. J Acquir Immune Defic Syndr (1999).

[15] Onono M (2014). HIV serostatus and disclosure: implications for infant feeding practice in rural South Nyanza, Kenya. BMC Public Health.

[16] Aluisio A (2011). Male antenatal attendance and HIV testing are associated with decreased infant HIV infection and increased HIV-free survival. J Acquir Immune Defic Syndr.

[17] McGrath CJ, et al. Non-disclosure to male partners and incomplete PMTCT regimens associated with higher risk of mother-to-child HIV transmission: a national survey in Kenya. AIDS Care. 2017:1–9. 10.1080/09540121.2017.1400642 PMC5895526 29130333

[18] National AIDS and STI Control Programme (NASCOP), K., *Kenya AIDS Indicator Survey* 2012*: Final Report* June 2014, DOI: 10.15226/sojmid/2/3/00122.

[19] Kiarie J (2012). National evaluation of PMTCT services; Kenya. *9th Conference on Retroviruses and Opportunistic Infections*. 2012. Seattle, Washington, march.

[20] Kinuthia J (2011). Uptake of prevention of mother to child transmission interventions in Kenya: health systems are more influential than stigma. J Int AIDS Soc.

[21] World Health Organization (2012). A short guide on methods: measuring the impact of national PMTCT programmes: towards the elimination of new HIV infections among children by 2015 and keeping their mothers alive.

[22] Sherin KM (1998). HITS: a short domestic violence screening tool for use in a family practice setting. Fam Med.

[23] Hayes-Larson E, et al. Prevalence, patterns, and correlates of HIV disclosure among TB-HIV patients initiating antiretroviral therapy in Lesotho. AIDS Care. 2017:1–7. 10.1080/09540121.2017.1280124 PMC5469711 28100068

[24] Roxby A (2013). Pregnant women and disclosure to sexual partners after testing HIV-1-seropositive during antenatal care. AIDS Patient Care ST.

[25] National AIDS Control Council, Kenya AIDS Response Progress Report 2014: Progress towards Zero 2014, DOI: 10.15226/sojmid/2/3/00122.

[26] NASCOP. Prevention Mother to Child Transmission 2012; Available from: http://guidelines.health.go.ke:8000/media/Guidelines_for_PMTCT_of_HIVAIDS_in_Kenya-1.pdf.

[27] NASCOP, National eMTCT communication strategy. 2012. Available from: https://www.thehealthcompass.org/project-examples/kenya-national-emtct-communication-strategy-2012-2015.

[28] Dunkle K (2008). New heterosexually transmitted HIV infections in married or cohabiting couples in urban Zambia and Rwanda: an analysis of survey and clinical data. Lancet.

[29] Mitsunaga TM (2005). Extramarital sex among Nigerian men Polygyny and Other Risk Factors. J Acquir Immune Defic Syndr.

[30] Maher D (2011). Concurrent sexual partnerships and associated factors: a cross-sectional population-based survey in a rural community in Africa with a generalised HIV epidemic. BMC Public Health.

[31] Kasamba I (2011). Extraspousal partnerships in a Community in Rural Uganda with High HIV prevalence: a cross-sectional population-based study using linked spousal data. JAIDS J Acquir Immune Defic Syndr.

[32] Larsson EC (2012). Opt-out HIV testing during antenatal care: experiences of pregnant women in rural Uganda. Health Policy Plan.

[33] Issiaka S (2001). Living with HIV: women's experience in Burkina Faso, West Africa. AIDS Care.

[34] Antelman G (2001). Predictors of HIV-1 serostatus disclosure: a prospective study among HIV-infected pregnant women in Dar es salaam, Tanzania. AIDS.

[35] Ramirez-Ferrero E, Lusti-Narasimhan M (2012). The role of men as partners and fathers in the prevention of mother-to-child transmission of HIV and in the promotion of sexual and reproductive health. Reprod Health Matters.

[36] Kiula E, Damian D, Msuya S (2013). Predictors of HIV serostatus disclosure to partners among HIV-positive pregnant women in Morogoro, Tanzania. BMC Public Health.

[37] Olagbuji BN (2011). Spousal disclosure of HIV serostatus among women attending antenatal care in urban Nigeria. J Obstet Gynaecol.

[38] Nkuoh GN, Meyer DJ, Nshom EM (2013). Women's attitudes toward their partners’ involvement in antenatal care and prevention of mother-to-child transmission of HIV in Cameroon, Africa. J Midwifery Womens Health.

[39] Maman S, Moodley D, Groves AK (2011). Defining Male Support During and After Pregnancy From the Perspective of HIV-Positive and HIV-Negative Women in Durban, South Africa. J Midwifery Womens Health.

[40] Shamu S (2011). A systematic review of African studies on intimate partner violence against pregnant women: prevalence and risk factors. PLoS One.

[41] Kiarie JN (2006). Domestic violence and prevention of mother-to-child transmission of HIV-1. AIDS.

[42] Makin J (2008). Factors affecting disclosure in south African HIV-positive pregnant women. AIDS Patient Care ST.

[43] Farquhar C (2004). Antenatal couple counseling increases uptake of interventions to prevent HIV-1 transmission. J Acquir Immune Defic Syndr.

[44] McGrath CJ (2018). Non-disclosure to male partners and incomplete PMTCT regimens associated with higher risk of mother-to-child HIV transmission: a national survey in Kenya. AIDS Care.

[45] Cooper ER, et al., Combination antiretroviral strategies for the treatment of pregnant HIV-1-infected women and prevention of perinatal HIV-1 transmission*.* J Acquir Immune Defic Syndr Apr 200215;29(5): p. 484–494. 10.1097/00126334-200204150-00009 11981365

[46] Farquhar C (2001). Partner notification by HIV-1 seropositive pregnant women: association with infant feeding decisions. AIDS.

[47] Kuonza L (2010). Non-adherence to the single dose nevirapine regimen for the prevention of mother-to-child transmission of HIV in Bindura town, Zimbabwe: a cross-sectional analytic study. BMC Public Health.

[48] Jasseron C (2013). Non-disclosure of a pregnant Woman’s HIV status to her partner is associated with non-optimal prevention of mother-to-child transmission. AIDS Behav.

[49] Baeten JM (2012). Antiretroviral prophylaxis for HIV prevention in heterosexual men and women. N Engl J Med.

[50] Weller S and Davis K, Condom effectiveness in reducing heterosexual HIV transmission. Cochrane Database Syst Rev. 2002;, 2002. 1(CD003255.), DOI: 10.1002/14651858.CD003255. 10.1002/14651858.CD003255 PMC8407100 11869658

